# Serious Games for Preventing Musculoskeletal Disorders in Occupational Settings: Scoping Review

**DOI:** 10.2196/66913

**Published:** 2025-10-21

**Authors:** Thomas Rivière, Thibaud Hulin, Farzan Sasangohar, Mohsen Zare

**Affiliations:** 1 ELLIADD (UR 4661) UTBM Université Marie et Louis Pasteur Belfort France; 2 ELLIADD (UR 4661) Université Marie et Louis Pasteur Montbéliard France; 3 Department of Industrial and Systems Engineering Texas A&M University College Station, TX United States

**Keywords:** musculoskeletal disorders, serious games, occupational health, prevention, exergaming, video games

## Abstract

**Background:**

Musculoskeletal disorders (MSDs) are a significant health concern in the workplace, and while ergonomic interventions are commonly used, their long-term effectiveness is often questioned. Serious games (SGs), designed to go beyond entertainment, have emerged as a promising tool that may address some of the limitations of traditional interventions, such as the need for sustained impact and greater worker engagement.

**Objective:**

This review aims to identify and analyze the key characteristics of SGs—including design, gameplay, and expected outcomes—that have been developed for the prevention or mitigation of work-related MSDs. Additionally, it explores the documented effects of SG implementation, assessing their potential contribution to MSD prevention and intervention strategies.

**Methods:**

A scoping review was conducted across 6 scientific databases (APA PsycInfo, Web of Science, Science Direct, MEDLINE, IEEE Xplore, and Google Scholar) to identify relevant studies published up to 2025. The selection process involved a multistep screening, including title and abstract review, followed by full-text assessment by 2 independent reviewers. Studies included were original research articles in English addressing MSD prevention and mitigation. Exclusions applied to studies on nonwork-related MSDs, limited content, duplicates, or repurposed entertainment games or gamification solutions. Data extraction was performed using a standardized form to capture key study characteristics. A 2-level analysis was applied: descriptive analysis, categorizing studies based on study characteristics and primary focus (design, evaluation, or both), and content-based analysis, examining game design, gameplay, expected outcomes, and evaluation methods to provide a structured synthesis of findings.

**Results:**

The initial search identified 2700 records, with 15 studies meeting the inclusion criteria. These studies explored diverse applications of SGs for MSD prevention, focusing either on game design and development or on educational impact assessment. Notably, only 2 studies comprehensively addressed both design methodology and educational evaluation. Findings revealed considerable variability in design approaches, technological platforms, gameplay mechanics, and expected outcomes. Additionally, the literature exhibited significant inconsistencies in evaluating SG effectiveness, with methodological limitations affecting comparability. While some studies targeted rehabilitation or occupational health and safety, only a few explicitly focused on MSD prevention, with a predominant emphasis on physical risk factors, whereas psychosocial and organizational aspects remained largely underexplored.

**Conclusions:**

This review highlights the need for standardized protocols and criteria for the design and evaluation of SGs to enable further synthesis and impact measurement. The integration of MSD prevention into SGs remains limited and is often approached indirectly through related themes such as workplace safety or rehabilitation. Future research should focus on developing and validating more comprehensive SG-based interventions and exploring their potential as effective tools in occupational health. The findings indicate a substantial gap in empirical evidence regarding the effectiveness of SGs for MSD prevention, largely due to the disparity in experimental approaches.

## Introduction

### Shifting MSD Prevention Toward Interactive Solutions

Work-related musculoskeletal disorders (MSDs) are a major public health issue and the leading cause of occupational illness in Europe, with incidence rates increasing [1,2]. The European Agency for Safety and Health at Work reports that MSDs account for 60% of work-related health issues in the European Union [1]. In 2019, more than 100 million European workers were affected, with back pain (43%) and neck or shoulder discomfort (41%) being the most common [1,3]. The prevalence is particularly high among sectors such as construction, water supply, agriculture, forestry, and fishing [1]. Data from the French social health insurance system estimates the economic burden of MSDs to be approximately 2 billion euros (US $2.35 billion) [4], contributing to significant labor force loss and financial strain. Intensified work conditions, task monotony, shortened cycles, and high psychological demands contribute to this trend [5,6]. Given the multiple risk factors, it is crucial to implement effective prevention strategies [7].

To address this growing concern, various preventive strategies have been developed. Effective interventions require a comprehensive approach that integrates technical solutions, organizational changes, and worker training [8-10]. Operator training is a widely adopted preventive measure aimed at increasing awareness, promoting behavior change, and supporting optimal work practices [11].

Numerous industries integrate MSD prevention programs into their operational frameworks, often including operator training. However, the implementation of such training varies across sectors [12] and faces challenges related to engagement, practical application, and knowledge retention [13]. Studies suggest that traditional training alone is insufficient for achieving sustained behavior change because of inconsistent implementation and a lack of standardization [7]. Moreover, the effectiveness of these programs remains uncertain, as many interventions are not systematically documented, and randomized controlled trials often fail to demonstrate a significant impact [11]. For instance, Hogan et al [14] found that manual material handling training did not significantly reduce MSD incidence, while Yu et al [15] concluded that training alone is insufficient for preventing MSDs. Even when training interventions show some efficacy [16], their long-term impact remains questionable [17].

While training programs and awareness campaigns are essential, they may not always ensure the long-term adoption of preventive behaviors, necessitating a paradigm shift. This shift involves using technologies to create immersive and interactive learning environments that are better aligned with workers’ realities. Among these technologies, serious games (SGs) offer a complementary approach to diversify prevention strategies and enhance existing interventions. Rather than replacing conventional methods, SGs capitalize on interactivity and engagement to reinforce key messages and promote behavioral change [18,19].

### Serious Games for Occupational Settings

To fully understand the role of SGs in occupational training, it is important to define their key characteristics. An SG is a computer application designed to integrate the characterizing goals [20] such as prevention, education, communication, or behavior change with the engaging features of video games [21]. SGs enhance engagement, sustain motivation, and create an environment where users can experiment, fail, and learn without real-world consequences [22-24]. This aspect is particularly relevant for MSD prevention, as it allows workers to explore risk situations and test alternative solutions in a controlled, risk-free space. Additionally, SGs offer flexibility in training schedules, allowing workers to engage in learning during their leisure time, which may contribute to higher adoption rates and sustained motivation [25-27].

Beyond their general benefits, SGs can also be tailored to meet the specific needs of different workplace environments. They can simulate realistic work settings in a way that personalizes the experience and may lead to enhanced performance outcomes [28]. These features are not inherent to SGs but can be incorporated depending on the target audience and intended outcomes. In MSD prevention, SGs can help workers recognize risk factors, understand ergonomic principles, and apply preventive strategies in their daily activities.

### Research Gap and Objectives

Despite their potential, the effectiveness of SGs in occupational settings remains uncertain. The difficulty in demonstrating their impact is largely due to methodological limitations that undermine the reliability of findings [29]. Material and software constraints also present challenges, potentially affecting implementation and adoption. Additionally, a misalignment between game mechanics and intended outcomes may diminish the educational and preventive value [30]. While gamification can produce notable cognitive outcomes, its impact on motivation and behavior is inconsistent. In particular, extrinsic motivation mechanisms can sometimes overshadow intrinsic engagement, reducing overall effectiveness [31].

Given these uncertainties, a scoping review of the literature is essential to understand how SGs have been designed and evaluated for MSD prevention. As a preliminary step to investigate this gap, this study aims to identify the key characteristics of SGs—including design, gameplay, and expected outcomes—used to address MSD prevention. Additionally, it explores whether any observable effects related to SG implementation have been documented.

## Methods

### Study Design

This study follows a scoping review approach, adhering to the methodological framework of Arksey and O’Malley [32] and further refined by Levac et al [33]. The findings are reported in accordance with the PRISMA-ScR (Preferred Reporting Items for Systematic Reviews and Meta-Analyses extension for Scoping Reviews) checklist (Multimedia Appendix 1) [34].

### Search Strategy

A systematic literature search was conducted in APA PsycInfo, Web of Science, Science Direct, MEDLINE via PubMed, and IEEE Xplore. Additionally, Google Scholar was searched, and ResearchRabbit was used to identify further relevant studies. The search covered all studies published up to 2025. The search strategy combined terms related to MSD prevention and SGs: (“musculoskeletal disorders” OR “musculoskeletal injury” OR “musculoskeletal disease” OR “MSD” OR “repetitive strain injury” OR “cumulative trauma disorder” OR “occupational health” OR “occupational safety” OR “occupational prevention” OR “occupational disorder” OR “ergonomic” OR “prevention” OR “risk reduction” OR “workplace intervention”) AND (“serious game” OR “game-based” OR “educational game” OR “digital game” OR “learning game” OR “prevention game” OR “digital educational game” OR “video game” OR “gamification” OR “game” OR “gaming”).

Search queries were tailored to each database’s syntax (eg, title or abstract in MEDLINE, topics in Web of Science, and abstract or full text in IEEE Xplore). Detailed search queries are provided in Multimedia Appendix 2.

### Eligibility Criteria and Study Selection

To ensure the selection of relevant studies, specific inclusion and exclusion criteria were established based on the scope of this review. These criteria are outlined in Textbox 1.

Studies on occupational safety management were included if they addressed MSD prevention.

**Eligibility criteria.**

**Inclusion criteria**

- Written in English and published as original research articles (excluding review articles and meta-analyses).
- Addressed musculoskeletal disorder (MSD) prevention, rehabilitation, addressing work-related MSDs, or occupational safety.
- Considered at least one aspect of MSD prevention, including biomechanical, organizational, or psychosocial factors.
- Investigated serious games explicitly designed for MSD-related prevention or training.

**Exclusion criteria**

- Focused on musculoskeletal conditions unrelated to work (eg, sports injuries, nonwork-related rehabilitation, and general physiology).
- Had limited content (eg, posters and short conference abstracts).
- Were duplicate publications from the same project (only the most comprehensive and recent version was retained).
- Examined entertainment video games repurposed for training or gamification solutions (ie, game mechanics applied in nongame contexts [35]) as these do not meet the strict definition of serious games.

### Study Selection Process

A rigorous multistep selection process was implemented to ensure methodological robustness and transparency (Multimedia Appendix 3).

#### Title and Abstract Screening

One reviewer (TR) initially screened all retrieved records (n=2700) to exclude studies that did not meet the inclusion criteria. The remaining studies were then compiled into a structured database, documenting key bibliographic details for systematic evaluation.

#### Full-Text Screening and Eligibility Assessment

Following title and abstract screening, 2 independent reviewers (TR and MZ) conducted a full-text assessment of the remaining studies to determine their eligibility. In cases of disagreement, a third reviewer provided arbitration to reach a consensus. To enhance efficiency and ensure consistency in decision-making, Rayyan software [36] was used for study selection.

#### Final Study Selection

All eligible studies underwent a final review before inclusion in the analysis. The overall selection process is presented as a flow diagram in the Results section.

### Data Charting Process and Analysis

A standardized data-charting form was used to systematically extract relevant study characteristics, including publication details (year and country, study design, and target populations), MSD approach (biomechanical, psychosocial, or organizational), type and description of the serious game (utilitarian objective, gameplay, and expected outcomes), and experimental methodology (assessment measures design, experimental methodology, and platforms and control devices of games; Multimedia Appendix 3).

To systematically examine the focus and contributions of the included studies, a 2-level analytical approach was used. The first level focused on general study characteristics, including publication year, discipline, study design, and target populations. It also categorized studies based on their primary objective. This level of descriptive analysis is outlined in Textbox 2.

The second level of analysis examined the core components of SG development and evaluation through 5 key parameters. To establish these parameters, we analyzed commonalities and divergences across studies regarding SG development, deployment, and assessment. Thematic coding revealed 5 recurring dimensions listed in Textbox 3.

**Descriptive analysis.**

- Design-focused studies: Research primarily investigating game development, technologies, and methodologies.
- Evaluation-focused studies: Research assessing the effectiveness, usability, and engagement of serious games in achieving intended outcomes [26].
- Combined design and evaluation studies: Studies integrating both game development and assessment of its impact.

**Content analysis.**

1. Design methodologies: These include collaborative, iterative, and participatory approaches, with a focus on technology-centered development.
2. Platforms and control devices: Games can be implemented on various platforms, including personal computers, consoles, mobile devices, and virtual reality systems. Control devices may involve haptic controllers, motion capture systems, and keyboards and mice.
3. Gameplay: Games vary across different categories such as simulations, exergames (games designed for physical exercise [37]), and management games. This feature encompasses mini-games, narrative elements, feedback systems, customization options, points and scoring, competition, and audio components.
4. Expected outcomes: The anticipated results include improvements in rehabilitation, heightened occupational health and safety awareness, musculoskeletal disorder prevention, user engagement, usability, user experience, and educational impact.
5. Evaluation methods: Evaluation methods assessed engagement, usability, user experience, and educational impact.

## Results

### Study Selection

**Figure 1:**
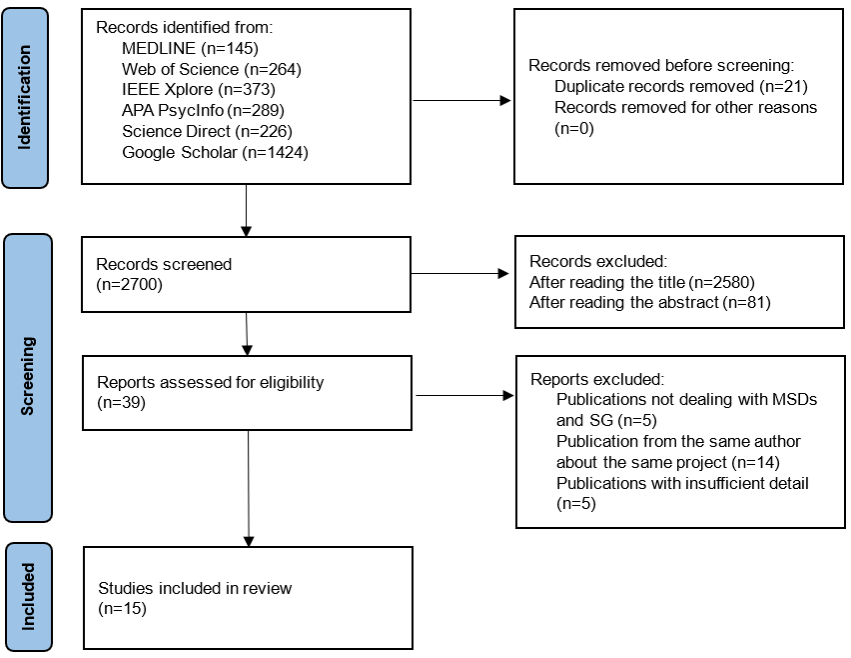
The PRISMA (Preferred Reporting Items for Systematic Reviews and Meta-Analyses) flow diagram of the selection process of the studies included in this review. MSD: musculoskeletal disorder; SG: serious games.

A total of 2700 records were initially retrieved. After title screening, irrelevant and duplicate records were removed, leaving 120 studies for further evaluation. Abstract screening reduced the selection to 39 eligible studies, and after full-text assessment, 15 studies met the predefined inclusion criteria. The data selection procedure is shown in Figure 1 using a PRISMA-ScR flow diagram.

### Characteristics of the Included Studies

Based on the first-level analysis, the included studies were published between 2013 and 2025. Geographically, studies were carried out in Europe (n=8), Asia (n=2), North America (n=2), South America (n=1), and Oceania (n=1). One study has a dual continental origin, the result of a collaboration between Colombian and Canadian researchers.

The studies addressed 3 primary application areas: rehabilitation SGs addressed work-related MSDs (n=4) designed for patients with MSDs [38-41], occupational health and safety games (n=5) aimed at enhancing risk management and injury prevention in workplace settings [42-46], and MSD prevention games (n=6), targeting physical risk factors and ergonomic training to mitigate occupational MSDs [47-52]. The games developed by Jansen-Kosterink et al [38] and France and Thomas [40], while not explicitly designed for occupational settings, were retained in our review due to their focus on chronic low back pain—one of the most prevalent forms of work-related MSDs. The target populations varied across studies: people with MSDs (n=4), health care professionals (n=1), industrial workers (n=3), including automotive industry workers (n=2) and watchmakers (n=1), sedentary office workers (n=2), high school students (n=1), children aged between 8 and 14 years (n=1), and unspecified target population (n=3). Across all studies, SGs were proposed as an alternative to traditional training methods, using their interactive and engaging nature to enhance learning and behavior change.

Additionally, the reviewed studies were further classified based on their primary focus on game design, evaluation, or both (Multimedia Appendix 4 [38-52]). Design-oriented studies (n=8) primarily investigated game development methodologies, with limited focus on evaluation. In most cases, evaluation was restricted to usability testing conducted at the final stage of game development [39,41,44-46,48,49,51]. These studies did not assess the long-term behavioral impact or MSD symptom reduction. Evaluation-oriented studies (n=5) conducted detailed assessments of SG usability, engagement, and effectiveness, but provided limited insights into game design methodologies [38,40,42,50,52]. Only 2 studies covering both design and evaluation comprehensively examined both development methodologies and games’ impact on behavioral change [43,47].

### Content Analysis of Game Design and Its Evaluation

#### Design Methodology

Among the design methods identified in the reviewed studies, collaborative approaches are predominant, with multiple stakeholders participating in various stages of game development. The collaborative design approach is prominently characterized by the involvement of various stakeholders, including end users, ergonomists, and game developers. This approach aligns with the principles of design-based research, as outlined by Sanchez and Monod-Ansaldi [53]. Design-based research emphasizes a systematic yet flexible methodology that seeks to enhance educational practices through iterative analysis, design, development, and implementation, driven by collaboration among researchers and practitioners within real-world contexts.

Three articles provided very limited details regarding their design methodology, concentrating almost exclusively on the technical characteristics of the game [39,41,51]. In these cases, the perspective appears to be more oriented toward demonstrating technological feasibility than toward adopting a participatory approach that incorporates the needs of end users. While such approaches may serve as exploratory or experimental objectives, they do not provide clear insight into how learning objectives, user needs, engagement strategies, and evaluation criteria were defined and integrated into the game, thereby limiting the understanding of whether the intended educational or behavioral outcomes were achieved.

Although not all studies explicitly detail their design methodologies, a common practice observed is the involvement of users in varying degrees and stages of the design process with an iterative approach. Several studies [39,43,45,49] have documented user involvement in the early stages of the development process. For example, Lanzotti et al [43] engaged users in shaping various design aspects of the game, such as character perspective, avatar visualization, and graphical detail. This user feedback, gathered from a sample of approximately 10 participants, guided design decisions. The optimal configuration was determined through a 10-point evaluation process. On the other hand, Rodrigues et al [48] and Idriss et al [39] included users primarily during the testing phase to evaluate whether the serious game met its basic functional requirements. However, their focus did not extend to aspects such as user experience or broader usability considerations.

The methods used to engage users in the design phase also exhibit notable diversity, with some studies using virtual simulations and structured feedback mechanisms. For example, Sisto et al [49] used a virtual simulation of decontextualized activities within the game that are conceptually similar to real-world work tasks. Ergonomics experts and game developers collaborated closely to define these game scenarios, using insights drawn from field data related to actual operational activities to ensure that the simulated activities effectively represent the types of tasks encountered in real work environments. Rapp et al [45] similarly integrated users into their game development process through a combination of semistructured interviews and web-based questionnaires. The data obtained helped specify the educational objectives of the game and refine the initial design elements.

While most included studies incorporate user input at specific stages, 3 studies integrate stakeholders throughout the entire development process, offering a more comprehensive co-design approach. Pietrafesa et al [44] used an advanced collaborative design approach that used both qualitative and quantitative methods. Users were engaged actively across various phases of the game’s development, including through surveys, focus groups, questionnaires, and discussions. User testing was conducted toward the end of the development cycle. Particularly, the authors sought input from both primary users and developers involved in the design process. Similarly, Kuipers et al [47] and Rebelo and Filgueiras [46] adopted an iterative design method grounded in collaboration between prospective users and experts. This method involved distinct stages: defining requirements, setting objectives and selecting technology, constructing prototypes, deploying the prototypes, evaluating them with target users, and conducting a comprehensive review by an expert panel.

Overall, the sophistication of the design methodologies appears to correlate with the effectiveness of the developed games. Games developed through advanced co-design methodologies often exhibit greater sophistication and functionality. Many researchers emphasize the importance of multidisciplinary and participatory collaboration in the development of SGs [53-55]. Hulin [56] underscores the specifics of the co-design approach within a research-action framework, introducing a novel method for analyzing design processes through stratigraphic modeling of experiences. Additionally, involving end users as consultants and testers—focusing their feedback on game dynamics—has been shown to enhance the game impact [57,58].

#### Platforms and Control Devices

To understand the technological landscape of SGs for MSD prevention, we examined the platforms and control mechanisms used in the reviewed studies. Among the reviewed studies, a variety of platforms and control mechanisms for SGs are evident. Four games were designed for personal computers (PCs) and use keyboard and mouse controls [42-45], while others apply haptic controllers [48] and cameras [41]. Three games were developed for smartphones and tablets [42,44,50]. Several games used motion-capture technologies such as Microsoft Kinect [39,47,48,52], inertial measurement unit (IMU) [51], Leap Motion [49], or similar tools like Vive Tracker [40]. One study used a more advanced motion capture system combining a reflective-marker suit, infrared cameras, and surface electromyography electrodes [38]. In some cases, the games used virtual reality (VR) devices [40,49] alongside motion-capture technologies, with the latter often serving as measurement tools. It is noteworthy that all the reviewed SGs were digital in format [59].

Sisto et al [49] used a combination of motion-capture technologies (Microsoft Kinect and Leap Motion) within a VR game. Their approach aimed to capture subtle hand movements with Leap Motion while using Microsoft Kinect to assess the positions of various body parts, including the back, arms, and neck. Similarly, Stranick and Lopez [52] used a VR headset in order to immerse the user in the game environment, leveraging Microsoft Kinect to adopt the expected posture for stretching exercises. Rodrigues et al [48] used multiple gaming peripherals, incorporating gestural control via Microsoft Kinect, and haptic devices. Their game architecture featured segments that are played on a PC with haptic controllers, while other segments used Kinect to detect when players needed breaks and to suggest exercises.

Some games, such as those developed by Greuter and Tepe [42] and Pietrafesa et al [44], privileged minimal hardware, designed specifically for tablets, smartphones, and PCs. The motivation for choosing minimal hardware requirements in game development often revolved around increasing accessibility, particularly for classroom settings or enabling students to use the game on their smartphones beyond class hours. However, the rationale behind specific sophisticated technology choices, such as VR, or haptic controllers, is rarely detailed in the reviewed studies. For example, Rodrigues et al [48] used haptic devices in their game, but the study lacks a clear justification for this choice, missing a clear explanation of the benefits and limitations of using haptic technology.

Games designed for rehabilitation, or the acquisition of physical skills, predominantly used motion capture technologies [38-40,47-49]. The choice of technology in these cases is closely tied to its functional utility within the game's context. However, the implementation of such technologies can introduce specific constraints. For example, in Valdivia et al [51], although the use of an IMU allows for faster sampling and more accurate capture, it is invasive and requires the expertise of specialists. Similarly, Stranick and Lopez [52] and Sisto et al [49] reported notable technical limitations associated with the use of VR technology. In Stranick and Lopez [52], the Microsoft Kinect sometimes failed to capture player movements in time. In Sisto et al [49], players had to keep their hands within their field of vision to avoid losing in-game items, and the VR system occasionally demonstrated suboptimal fluidity, requiring players to repeat certain actions. Additionally, the setup of the VR system was time-consuming and required the expertise of a VR specialist to address potential system bugs and ensure the game's proper functioning.

#### Gameplay

To clearly define the scope of gameplay in the reviewed studies, it is necessary to distinguish between its core components and related elements such as narrative scenarios. Here, “gameplay” is defined as the interactive and functional aspects of a game, encompassing its rules, mechanics, objectives, and the overall player experience. In contrast, a “scenario” refers to the narrative or storyline that provides the contextual framework for the game and structures the sequence of events during gameplay [59]. A single gameplay framework can support multiple scenarios.

The reviewed studies reveal a wide range of formats, from very simple structures to more elaborate ones. A first category of gameplay includes short and simple mini-games, based on basic interactions and often limited to a single task or objective [41,51,52]. A second category consists of relatively simple games that incorporate a progression or performance tracking system, allowing for a sense of continuity in the player experience [43,49,50]. A third configuration involves games composed of multiple successive mini-games, designed to diversify situations and gaming experiences [38-40,48]. The last category of gameplay consists of a more complex structure, combining various mechanics, levels of games, and integrating more advanced progression systems [42,44,45,47]. This diversity in gameplay reflects a variety of pedagogical intentions, as well as technical and contextual constraints specific to each project.

Several of the developed games show similarities in their underlying concepts and rules, while their distinctions appear at the level of narrative elements, artistic attributes, and control mechanisms. For instance, France and Thomas [40] and Jansen-Kosterink et al [38] both crafted memory-based games characterized by identical rule sets but differentiated by graphical elements and control mechanisms. Moreover, we observed games of similar genres, especially within the risk management category, within those designed for PCs [42,44,45].

In some PC-based games, player interaction extends beyond individual actions to managing environments and nonplayer characters (NPCs), adding a strategic component to gameplay. For example, in Pietrafesa et al [44], players are responsible for maintaining a safe work environment by overseeing NPCs' tasks and protecting them from workplace hazards. The player's success is tied to the productivity of the NPCs, which is affected by the implemented safety measures. Greuter and Tepe [42] require players to identify and address hazardous conditions affecting NPCs' construction work. Players mitigate these risks by providing necessary equipment and adjusting the work environment, enabling NPCs to continue their tasks. In Rapp et al [45], a multiplayer game simulates restaurant management, where players oversee various aspects such as personnel recruitment, budget management, and employee welfare. The game includes a debriefing phase aimed at enhancing learning outcomes, although details on the gameplay mechanics were not extensively provided in the study.

Some studies incorporated features designed to enhance player motivation and engagement, such as competition and collaboration [42-44,47]. Lanzotti et al [43] considered incorporating a shared scoreboard to introduce competition as a motivational element. Pietrafesa et al [44] added a competitive component by having 2 groups of students compete against each other, though the study does not provide detailed information on the conditions of this competition. Greuter and Tepe [42] and Kuiper et al [47] organized weekly group gaming sessions to facilitate testing, but this collaborative aspect was not further examined in the study.

Another critical aspect of gameplay complexity is the extent to which games integrate dynamic feedback and progression systems to reinforce learning objectives. Stranick and Lopez [52] incorporate elements such as achievements and ranking in their game, which are clear feedback and progression mechanisms for the player. Sisto et al [49] integrate task completion time and a posture risk assessment score as core gameplay elements. These scores are calculated using an algorithm that factors in exposure time and joint angles within the simulated environment. At the end of each game level, players can view their risk scores for awkward postures alongside their time performance scores, enabling them to refine their strategies for task execution within the game. In contrast, the game by Kuipers et al [47] is notably more sophisticated, incorporating elements of customization, scoring, and progression. Players are encouraged to explore the in-game world by visiting specific locations that trigger mini-games, which allow them to personalize the virtual environment. The primary objective of this game is to induce behavioral changes, motivating players to experiment with new behaviors while confronting challenging tasks and objectives. Throughout the gameplay, players receive precise and tailored feedback to ensure compliance with safety and in-game rules. Both the games developed by Kuipers et al [47] and Sisto et al [49] incorporate in-game experiences and dashboards with data visualization to support players in understanding and interpreting the information presented during gameplay.

Interestingly, despite the emphasis on interactivity, none of the studies, except for Greuter and Tepe [42], report considering audio elements and music in the game design which could influence player immersion and experience. In their study, participants provided negative feedback about the sound environment, which the authors hypothesized was due to all 20 games being played simultaneously in the same room, creating an overwhelming auditory experience. The authors suggest that the use of headphones might have mitigated this issue, although it could have also impeded player collaboration. Table 1 summarizes the technologies, gameplay, and expected outcomes used in the reviewed studies.

**Table 1:** List of studies according to platforms and control devices, gameplay, and expected outcomes of the game.

	Platforms and control devices	Gameplay	Expected outcomes
Jansen-Kosterink et al [38], 2013	Motion capture system (via suit, sensors, and infrared cameras)	Puzzle game and exergame: (1) control an avatar's movement on an obstacle course by walking pace, (2) control avatar's climb progress by raising arms, and (3) memorize and replicate a neck stretching movement sequence	Physically train patients with chronic neck and back pain through gameplay
Idriss et al [39], 2017	Microsoft Kinect	Exergame: (1) kick a ball after positioning correctly and (2) move an object between two tables	Make rehabilitation more engaging and fun for patients with MSDs^a^
France and Thomas [40], 2018	VR^b^ and motion capture system (Vive Tracker)	Puzzle game and exergame: (1) memorize a sequence of icons and replicate it by selecting icons through gestures and movements, (2) catch fish with a certain gesture and avoid shark attacks by ducking, and (3) dodge or catch balls thrown by characters (dodgeball)	Reduce fear and perception of pain and increase physical activity in patients with chronic back pain
Husna et al [41], 2025	PC^c^ and webcam	Exergame: making hand gestures to get a character out of a maze	Accompanying the rehabilitation work of people with MSD
Greuter and Tepe [42], 2013	iPad and PC (mouse)	Management game: identify hidden risks in a scene and propose corrective measures via an icon bar to allow NPCs^d^ to work on building a tower	Engage learners in OHS^e^ training and teach danger management procedures
Lanzotti et al [43], 2019	PC game (keyboard and mouse)	Simulation game: solve an action sequence by selecting the right tool and PPE^f^ and select the necessary actions to control a control panel	Train industrial operators in safety measures and practices for specific, personalized, and hazardous activities
Pietrafesa et al [44], 2021	Web-based game accessible on PC, tablet, and mobile	Management game: manage a healthy work environment by organizing work tasks and protecting characters via PPE	Raise awareness among high school students in vocational schools about health and safety at work
Rapp et al [45], 2019	PC	Management game: a multiplayer simulation game where players must manage a restaurant as executive managers	Provide understanding of occupational health and promote employee culture among the participants
Rebelo and Filgueiras [46], 2012	*Not documented*	*Not documented*	Raising young people's awareness of occupational health
Kuipers et al [47], 2016	Microsoft Kinect	Exergame: (1) catch sheep based on their proximity to the player and (2) maintain an orchard by harvesting fruits and watering trees	Instruct health care staff on lifting and transfer techniques
Rodrigues et al [48], 2018	Kinect, haptic controllers, and PC game	Exergame: (1) adapt the workspace by interacting with environmental elements to recreate a healthy environment for characters, (2) complete stretching exercises by moving puzzle pieces, and (3) replicate and hold the posture of a silhouette displayed on screen	Raise awareness of proper office work postures and stretching exercises
Sisto et al [49], 2018	VR and motion capture system (Kinect and Leap Motion)	Puzzle and simulation game: place gears in a specific arrangement (puzzle)	Train operators to find and practice the best postures and movements in their work activities
Intipanya et al [50], 2025	Smartphone with camera	Exergame: perform head stretching movements to control aircraft	Preventing cervical pain
Valdivia et al [51], 2017	Kinect and IMU^g^	Exergame: perform bending and extension movements to “paint” a room	Promote stretching exercises for office workers
Stranick and Lopez [52], 2022	Oculus VR and Kinect	Exergame: successively reproduce postures to cross obstacles	Promote stretching exercises for workers in industry

^a^MSDs: musculoskeletal disorders.

^b^VR: virtual reality.

^c^PC: personal computer.

^d^NPCs: nonplayer characters.

^e^OHS: occupational health and safety.

^f^PPE: personal protective equipment.

^g^IMU: inertial measurement unit.

#### Expected Outcomes

Defining the expected outcomes and learning objectives of SGs is crucial, as they specify the skills, knowledge, and competencies that developers aim for users to acquire. For SGs designed for the rehabilitation of patients with work-related MSDs [38-41], the scenarios are structured around movements and postures critical for recovery or training. The expected outcome is to engage patients in performing rehabilitation exercises through play scenarios and to help them overcome fear of injury through interactive participation. An example of a rehabilitation-focused SG is the VR-based intervention developed by France and Thomas [40], designed to gradually improve lumbar flexion. While the game may not directly influence psychological factors for behavior change, it serves as a distraction from pain, reinforcing movement by increasing expectations of lumbar flexion. This approach helps individuals cope with movement-related fear, promoting the understanding that lumbar flexion is not inherently harmful, particularly for those with chronic low back pain and pain-related fear.

The learning outcomes of the games addressing occupational health and safety [42-45] revolve around raising awareness and facilitating learning. These studies typically focus on the risks of work-related accidents, particularly safety procedures and equipment. For instance, the game developed by Greuter and Tepe [42] aligns with occupational health and safety training objectives, aiming to educate users on hazard identification, control measures, communication processes, and reporting procedures. However, the study did not assess whether playing the game improves students' ability to identify occupational hazards. Moreover, Rapp et al [45] expanded this approach by integrating broader workplace learning objectives, aiming to enhance leadership, communication, and safety culture. For example, they aim to enhance knowledge by involving employees in operational decision-making, develop skills by allowing players to explore various actions with feedback, and promote awareness and attitude shifts toward workplace health and safety by emphasizing its importance for both employee well-being and organizational success. However, the authors did not provide any results demonstrating the achievement of these objectives through their game.

Finally, a distinct category of SGs focuses on MSD prevention by training professionals to adopt proper postures and ergonomic behaviors [46-52]. Rodrigues et al [48] developed 3 complementary mini-games: one is designed to raise awareness about the risks associated with static desk postures, while the other two encourage players to engage in regular stretching exercises. Kuipers et al [47] and Sisto et al [49] designed games aimed at promoting behavioral changes, providing tailored feedback to support the effective adoption of new ergonomic practices. Within this category, three studies [50-52] specifically used exergames to promote stretching and movement exercises. Intipanya et al [50] created a smartphone-based exergame where head movements control an aircraft, targeting cervical pain prevention. Valdivia et al [51] used Kinect and IMU sensors to prompt office workers to perform bending and extension movements through a room painting task. Stranick and Lopez [52] designed a VR-based exergame where players reproduce postures to overcome obstacles, promoting stretching exercises among industrial workers. The exergames reviewed primarily focus on the correct execution of physical exercises, with short-term learning objectives centered on performance. Players’ cognitive involvement remains limited: few games encourage understanding the purpose of movements, reflecting on posture, or transferring learning beyond the game context. Moreover, game mechanics often emphasize repetition or movement accuracy without providing elements that help players understand their relevance or their connection to MSD prevention. The game included in Kuipers et al [47], however, goes further by promoting behavioral change among health care workers, particularly in patient handling practices.

#### Evaluation Methods

According to Gris and Bengston [60], the assessment of game-based learning effectiveness revolves around the key components including engagement, user experience, usability, and educational impact. The evaluation uses a combination of quantitative and qualitative measures for assessing educational impact, with qualitative methods being more commonly used for evaluating engagement, user experience, and usability [60]. In medical literature, another prevalent approach is to assess a game's ability to facilitate skill transfer [61]. The following section examines how different included studies evaluated various components of game effectiveness.

#### Engagement, Usability, and User Experience

The assessment of engagement, usability, and user experience is evident in 10 of the studies reviewed [38,39,41-43,46-48,51,52], although these evaluations exhibit variations in the questionnaires, variables, and methodologies used. Among these studies, some focused more specifically on user experience, using distinct evaluation methods and tools. Greuter and Tepe [42] evaluated participants' user experience by a questionnaire across various dimensions, including fun, engagement, success, control, motivation, feedback, usability, and difficulty [62]. Additionally, semistructured interviews were conducted following the test sessions to gather further insights. While the authors reported satisfactory levels of motivation, engagement, and learning among participants, with most students indicating increased motivation as they progressed through the game, several limitations in the study's design should be acknowledged. These include a small sample size, the subjective nature of the evaluation (which relied solely on self-reported questionnaires from a single group), and the absence of a longitudinal follow-up period.

Similarly, Jansen-Kosenterink et al [38] adopted a multifaceted approach to evaluate user experience, incorporating standardized and customized assessment tools. In their study, user experience was evaluated using multiple metrics: usability was measured with the System Usability Scale (SUS) [63], satisfaction was gauged through 2 specific questions, motivation was assessed using 2 additional questions, and gaming experience was evaluated with the Core Elements of Gaming Experience Questionnaire [64]. All 10 participants in the study reported a positive user experience, indicating that the game effectively distracted them from pain and enhanced their focus on gameplay. This study addressed the longitudinal aspects by tracking participants over a 4-week period of gameplay. However, the study's limitations include the absence of a control group and a relatively small sample size. Similarly, Husna et al [41] used the SUS and the User Experience Questionnaire to assess their game, although the choice of these tools is questionable given the simplicity of the prototype, in which a character is guided through a maze using hand gestures captured via video. The minimal graphics and interactive complexity of the system raise concerns about the actual relevance of evaluating usability and user experience in this context.

Some studies focused more on usability assessment, with Lanzotti et al [43] evaluating their SG based on effectiveness, efficiency, and satisfaction. Effectiveness and efficiency were measured using quantitative metrics, including interaction errors, incorrect movements, gameplay duration, and task completion rates, while satisfaction was assessed through a subjective questionnaire. The usability index was calculated as the average of these 3 factors. The study involved 2 groups of 6 operators: the case group, which used an SG for training on mould press safety procedures, and the control group, which completed a written test with a similar structure. Although the test group reported higher satisfaction, their overall usability index was lower, particularly in correctly sequencing commands for safe machine operation. Interpretation of the results was challenging due to the small sample size and the lack of information on participants' baseline knowledge levels. The authors suggested that the written test may have assessed theoretical knowledge, whereas the SG required practical application, potentially leading to discrepancies. Additionally, no weighting was applied to usability factors in calculating the index, and the questionnaire lacked details on reliability and validity. Furthermore, the study did not specify how variables such as error frequency were quantified, particularly for the control group, limiting the clarity of the findings.

In addition to evaluating usability, some studies also explored user preferences and subjective satisfaction. Rodrigues et al [48] assessed user preferences among 20 participants, divided into 2 age groups, focusing on 5 key aspects: game relevance, ease of use, effectiveness of stretching exercises, perceived health (before and after the session), and overall satisfaction. Participants rated each aspect on a 3-point scale (low, medium, and very good). Most participants rated the game as “very good” or “medium” across the 5 categories. However, fewer than 40% rated the effectiveness of stretching exercises as “very good,” and only about half gave the highest rating for overall satisfaction. This suggests that while participants found the game engaging, its perceived effectiveness in promoting stretching exercises was more variable. A key limitation of this study is the lack of details on the validity, reliability, and development of the assessment questionnaire. The absence of such information raises concerns about the robustness of the evaluation method, as it is unclear whether the tool effectively captured user perceptions and game impact.

Other studies further investigated usability and acceptability through comparative analysis between healthy participants and those with MSD symptoms. In that respect, Idriss et al [39] conducted an evaluation of the usability and acceptability of a serious game that featured 2 distinct scenarios: football and object manipulation. Feedback was collected through a questionnaire. The study also involved a comparison of game scores between 2 groups: healthy participants and those with MSD symptoms. The results indicated that healthy participants achieved significantly higher scores than those with MSD symptoms. Participants were asked to rate various parameters, including the clarity of objectives, level of difficulty, and the attractiveness of the game environment, using a 5-point Likert scale. Both groups found the games to be motivational, attractive, and challenging. However, the study did not include a comparative analysis of the usability results between the 2 groups. Additionally, the questionnaire used for evaluation had not been validated for reliability or validity, raising concerns about whether it adequately captured all relevant aspects of usability, engagement, and playability in the games.

Finally, two studies focused on the assessment of engagement. Valdivia et al [51] mainly assessed exergame’s engagement skills through the Game Engagement Questionnaire, which measures dimensions such as immersion, focus, and sense of control. This evaluation followed two brief 1-minute exercise sessions in which participants performed trunk flexion and extension movements using 2 versions of the exergame—one based on the Kinect V2 sensor and the other using an IMU. Despite the use of a standardized questionnaire, such short-duration exposure with a small subject raises questions about the ability of the sessions to foster meaningful engagement or allow users to fully grasp the potential of the system. While most studies relied on self-reported measures, Kuipers et al [47] introduced a different approach by quantifying engagement through voluntary re-engagement metrics. They used a quantitative measure for evaluating player engagement, where the number of game sessions voluntarily conducted by players over a given period served as an indicator of their engagement. This approach is analogous to the voluntary reengagement observed in Sanchez et al [65], where players' self-initiated participation in the task was used as a measure of motivation. During the experimentation phase, participants were given free time in which they could choose to replay the game at their discretion. The decision of a participant to replay the game independently was interpreted as a sign of motivation. However, Sanchez et al [65] emphasized that this measure is more closely associated with intrinsic motivation rather than engagement. This distinction between the two concepts should be considered in the interpretation of results.

In summary, in line with Gris and Bengston [60], the assessment of engagement, usability, and user experience in SGs predominantly relies on qualitative measures. Moreover, the evaluation tools used in these studies are generally nonstandardized. Among the studies reviewed that assess these concepts, only three [38,41,51] used a standardized questionnaire. Furthermore, there are notable discrepancies in the concepts and variables measured across different studies. The concept of usability, for instance, is variably defined depending on the study. For example, in the study of Lanzotti et al [43], usability encompasses a range of quantitative and qualitative measures, focusing on the dimensions of effectiveness, efficiency, and satisfaction.

#### Educational Impact of Serious Games

Assessing the educational impact of SGs requires robust evaluation methodologies to determine their effectiveness in training and behavior change. However, many studies fail to implement comprehensive assessment processes, relying instead on limited or inconsistent evaluation methods. Among the studies included in the analysis, many lack a comprehensive process for evaluating the impact of developed SGs on training or achieving targeted outcomes. Three reviewed studies [44,45,49] do not provide any evaluation of the game's impact on learning, while others rely solely on postgame questionnaires without including a control group or had significant limitations. For instance, Lanzotti et al [43] assessed the efficacy of game-based learning by comparing in-game metrics with responses from a control group to a multiple-choice questionnaire addressing the same simulated process. However, the level of training received by the control group was not clearly defined, and the results indicated that the control group outperformed the experimental group, suggesting the serious game's effectiveness was limited. Similarly, Greuter and Tepe [42] evaluated the game's effectiveness through a written questionnaire on covered concepts. Since the game aimed to enhance hazard identification skills, effectiveness was measured through a written test requiring participants to identify risks from photographs. Educational impacts were assessed by comparing pretest and posttest questionnaire results of the experimental group. Although the authors mentioned the presence of a control group, results between the two groups were not provided, and the overall study design lacked robustness due to the use of a validated questionnaire and postsession data only.

However, 3 studies implemented game evaluations through prolonged test sessions involving multiple groups and clear, comparable measurement variables. Kuipers et al [47] measured the evolution of participants' game scores based on the number and duration of sessions over a period of 3 weeks. They observed that the more players played, the higher the scores increased. The increase in scores thus reflects the effectiveness of the game: players autonomously exhibit the desired behavior. The authors posited that performing in-game movements mirrors actual workplace lifting techniques, thereby increasing game scores leading to training outcomes aligning with desired behavioral changes in lifting techniques. Nevertheless, further validation is required through longitudinal studies assessing behavioral changes in real workplace settings.

Similarly, Jansen-Kosterink et al [38] evaluated learning outcomes by analyzing motor skill improvements and clinical indicators before and after gameplay sessions. The game was administered to a cohort of patients afflicted with chronic neck and back pain, who engaged with the game over a 4-week duration. Among the parameters measured, including walking velocity, reach height, cervical range of motion, pain intensity, and pain disability, only cervical range of motion exhibited a significant increase over the testing period. Although the authors asserted that patients generally exhibited faster walking speeds, increased reach, and enhanced neck mobility after 4 weeks of gameplay, these results did not reach statistical significance, likely due to the limited sample size and the short duration of the testing period.

France and Thomas [40] conducted a 9-week clinical trial to evaluate their VR-based rehabilitation game for pain management and functional recovery. They assessed patient-reported disability, pain levels, spinal condition, and pain expectations, with follow-up monitoring over 48 weeks. Results showed that the experimental group experienced greater pain and disability reduction, along with decreased pain expectancy and improved lumbar flexion compared to the control group. These findings suggest that the VR game may help individuals with low back pain and movement-related fear engage in repeated sessions, promoting graded spinal motion.

## Discussion

### Challenges and Perspectives for Serious Games in MSD Prevention

The objective of this review was to identify and analyze the key characteristics of SGs developed for work-related MSD prevention and mitigation while exploring their potential impact. However, the lack of empirical evidence and methodological inconsistencies across studies make it challenging to conclusively determine the effectiveness of SGs in reducing MSDs. One major challenge is the inconsistency of experimental protocols, particularly due to variability in measurement methods across studies. While certain studies, particularly in the medical field, rely on detailed and robust experimental protocols (randomized controlled trials), others often suffer from methodological limitations such as small sample sizes and weak study designs lacking control groups. This is in line with the findings of Steiner et al [66] in their scoping review focusing on gamification in shoulder rehabilitation, highlighting significant methodological limitations across studies, including small sample sizes, a lack of control groups, and a lack of robust experimental designs. Such limitations are also identified in previous studies [67,68] and appear to be a key factor contributing to the inconsistency of results we documented across SG studies. Ideally, a comparison of 3 conditions—training or learning sessions, gaming sessions, and combined training or learning and gaming sessions—would have provided valuable insights, yet such comparisons were often unfeasible in the studies included.

Beyond experimental limitations, the diversity of research objectives and methodological frameworks further complicates the synthesis of convergent knowledge related to SGs developed for occupational health objectives. Understanding the educational impact of these games necessitates a thorough contextualization of the existing games [69]. The variability in the characteristics of SGs—including aspects such as design, gameplay, pedagogical objectives, target population, and context of use—further complicates this endeavor. A review of SGs in health professions education [70] found similar limitations and challenges in drawing conclusions about SGs' efficacy.

Additionally, the complexity of evaluating SGs is exacerbated by the difficulty in quantifying learning outcomes and behavioral changes over time, particularly outside the realm of exergames, where quantitative and physiological data cannot be used for measurement. A systematic review focusing on digital SGs in nursing education highlighted the scarcity of data on sustained behavioral outcomes and the need for more rigorous and longitudinal research designs [71]. Another review highlighted the importance of integrating behavioral change theories into game design to effectively measure and achieve desired health outcomes [72].

Moreover, none of the studies reviewed have considered users' level of experience with video games or their gaming culture. Previous studies on behavior change in persuasive health games found that participants' engagement and outcomes were significantly affected by their familiarity with gaming and concluded that users' gaming backgrounds should be considered to tailor games [73]. Silva et al [74] highlighted instances where participants easily navigated game challenges due to their regular engagement with video games.

Another challenge lies in the simplistic design of many SGs, which often fail to balance pedagogical objectives with engaging and immersive gameplay elements. As seen in studies such as Pietrafesa et al [44], game scenarios do not replicate pedagogical objectives effectively. As a result, these games often lack the critical ludic quality essential for maintaining player engagement [75]. A systematic review by Ambros-Antemate et al [76] showed the absence of iterative design and testing phases resulting in games that may not effectively meet user needs or learning objectives. Garske et al [77] examined SGs for prosthetic training and identified similar design shortcomings—such as the lack of user-centered design, oversimplified mechanics, and misalignment with learning objectives—that align with the results of our study. The success of a serious game depends on effectively balancing ludic elements with characterizing goals [78]. When this balance is disrupted, players may rapidly lose interest and motivation. Consequently, even SGs that are content-rich and technically sound may fail to achieve their intended outcomes if the ludic aspects are not sufficiently developed to maintain long-term user engagement [79]. Previous research has shown that the degree to which users find a game to be enjoyable is related to their motivation levels, leading to better engagement and learning outcomes [80].

To enhance educational impact, several researchers [45,68,81] argue for the necessity of debriefing in SGs, highlighting its potential to enhance understanding of game content, clarify misconceptions, and internalize important lessons. This reflective process not only facilitates immediate learning but also contributes to long-term retention of knowledge and skills acquired during gameplay. For example, although not directly addressing MSDs, Michelet et al [82] found that participants who engaged in a structured debriefing session postgameplay demonstrated better knowledge retention and skill acquisition compared to those who did not. However, debriefing remains largely underexplored in the reviewed SGs on MSD prevention.

Moreover, the emphasis of existing research remains primarily on rehabilitation and occupational safety, with limited focus on the direct prevention of MSDs. A limited number of studies [47-52] specifically address the topic of MSD prevention, which primarily focused on physical ergonomics or manual material handling. These results underscore the critical need for further research to explore additional facets of MSD prevention, such as organizational and psychosocial factors, within the design framework of SGs.

Despite the variability in objectives, design methodologies, technologies, and contexts, which complicates the synthesis of results, this literature review underscores several key challenges that future research on the design and evaluation of SGs for the prevention of MSDs must address. A critical area for improvement is the detailed documentation of user involvement in the design process, as this engagement is vital for promoting both the acceptance and effectiveness of these games. The debriefing phases and evaluations of engagement and usability must be rigorously formalized to ensure more consistent and reliable outcomes. Future studies must overcome existing methodological limitations by developing standardized evaluations to ensure the empirical validation of serious game interventions. It is also essential to broaden the scope of research beyond rehabilitation and safety to encompass the prevention of MSDs. This expansion should include a focus on the organizational and psychosocial dimensions of MSDs, using SGs to develop more comprehensive and effective interventions.

Therefore, we recommend adopting design methodologies that integrate multifactorial MSD prevention models, while ensuring that technological implementations remain practical and adaptable to real-world industrial settings. Strengthening evaluation frameworks, particularly in measuring long-term effectiveness, is also crucial for establishing SGs as viable intervention tools in occupational health.

### Limitations

This study has several limitations. First, the final selection includes a relatively small number of studies, primarily due to the specialized nature of the research topic and the selection methodology, which inherently constrained the available literature. Second, grey literature, including unpublished studies and industry-based reports, was excluded, potentially omitting relevant work on SGs for MSD prevention.

Furthermore, to maintain a balance between comprehensiveness and relevance, search queries were adapted to the specific constraints of each database. In some cases, broad terms such as “game” and “occupational” were limited to titles or combined with more specific qualifiers (eg, “health” and “disease”) to reduce the retrieval of irrelevant results. While necessary, these refinements may have inadvertently restricted the scope of the search.

To mitigate these limitations, 2 independent authors (TR and MZ) conducted the search in several stages of study, and in addition to querying major scientific databases, we also searched Google Scholar and ResearchRabbit to ensure the inclusion of all relevant studies.

### Conclusions

This review underscores that SGs for MSD prevention developed through collaborative and iterative design processes, particularly those involving end users and domain experts, tend to exhibit greater pedagogical and functional robustness, whereas limited stakeholder involvement often results in narrower and less effective outcomes. The technological landscape of these SGs is diverse, ranging from basic PCs to advanced VR and motion-capture systems; however, most studies do not provide clear justifications for their technology choices. Learning objectives are frequently limited to task execution, with minimal emphasis on reflective learning or transfer beyond the game. Moreover, the expected outcomes predominantly address physical ergonomics, often neglecting psychosocial and organizational contributors to MSD risk. The evaluation of SG effectiveness remains inconsistent, with many studies lacking methodological rigor or long-term assessment, and while some report gains in knowledge or behavior, the evidence base remains limited and fragmented, especially regarding sustained health improvements.

## Appendix

Multimedia Appendix 1 PRISMA-ScR checklist.

Multimedia Appendix 2 Search queries.

Multimedia Appendix 3 Detailed screening.

Multimedia Appendix 4 Studies classification.

## Abbreviations

IMU: inertial measurement unit
MSD: musculoskeletal disorder
NPC: nonplayer character
PC: personal computer
PRISMA-ScR: Preferred Reporting Items for Systematic Reviews and Meta-Analyses extension for Scoping Reviews
SG: serious game
SUS: System Usability Scale
VR: virtual reality
