# Supplementary material for: Serious Games for Preventing Musculoskeletal Disorders in Occupational Settings: Scoping Review
Source: JMIR Serious Games. 2025 Oct 21;13:e66913. doi: 10.2196/66913 (PMC12587018; doi:10.2196/66913)
Supplement: Multimedia Appendix 2 [file games_v13i1e66913_app2.docx]

Search Queries

# Google Scholar

## Search

("musculoskeletal disorder" OR "musculoskeletal injury" OR "musculoskeletal disease" OR MSD OR "repetitive strain injury" OR "cumulative trauma disorder" OR "occupational health" OR "occupational disease" OR "occupational injury")

AND (prevention OR "risk reduction" OR "workplace intervention" OR ergonomic)

AND (intitle:game OR "serious game" OR "educational game" OR "digital game" OR "game-based" OR gamification)

## Results

1420 (17/03/2025, without citations)

## Caption

intitle: = looking in the title only

# MEDLINE

## Search

(("serious game*"[Title/Abstract] OR game*[Title/Abstract] OR gami*[Title/Abstract])

AND ("musculoskeletal disorder*"[Title/Abstract] OR "musculoskeletal injur*"[Title/Abstract] OR "musculoskeletal disease*"[Title/Abstract] OR "repetitive strain"[Title/Abstract] OR"cumulative trauma disorder*"[Title/Abstract] OR " occupational health" [Title/Abstract] OR "occupational disease" [Title/Abstract] OR "occupational injury" [Title/Abstract])

AND (english[Filter])) NOT (review[Publication Type])

## Results

142 (17/03/2025, 181 with review)

## Caption

[Title/Abstract] = search in title and abstract only.

[All Fields] = search in all fields.

[Publication Type] = publication type.

# Web of Science

## Search

(TS=("serious game" OR game* OR gami*))

AND TS=("musculoskeletal disorder*" OR "musculoskeletal injur*" OR "musculoskeletal disease*" OR "repetitive strain" OR "cumulative trauma disorder*"

OR "occupational health" OR "occupational disease*" OR "occupational injur*")

+ English filter

## Results

## 306 (314 without English filter), 264 (without magazine article, on 18/03/2025)

## Caption

TS = Searches title, abstract, keyword plus, and author keywords.

# IEEE Explore

## Search

("All Metadata":"serious game*"OR"game*"OR"gami*")

AND ("All Metadata":"musculoskeletal disorder*"OR"musculoskeletal injur*"OR "musculoskeletal disease*"OR"repetitive strain"OR"cumulative trauma disorder*" OR "All Metadata":occupational*)

## Results

## 373 (371 with the no review filter, on 18/03/2025)

## Caption

All Metadata = Includes the abstract, index terms, and bibliographic citation data (such as document title, publication title, author, etc.)

# APA PsycInfo

## Search

AB (("serious game*" OR (game*) OR (gami*)) AND AB (((musculoskeletal W2 injur*) OR (musculoskeletal W2 disorder*) OR (musculoskeletal W2 disease*) OR (repetitive W2 strain W2 injur*) OR (cumulative W2 trauma W2 disorder*) OR (occupational*))

## Results

289 (on 18/03/2025)

Caption

W2 means that the words must appear within 2 words of each other, in the order specified. Only works for APA PsycInfo.

AB = Abstract

# Science Direct

## Search

Title, abstract, keywords: (musculoskeletal OR "repetitive strain" OR "cumulative trauma" OR occupational) AND ("serious game" OR game OR gamification)

## Results

226 with English filter (on 18/03/2025)
