# Supplementary material for: Serious Games for Preventing Musculoskeletal Disorders in Occupational Settings: Scoping Review
Source: JMIR Serious Games. 2025 Oct 21;13:e66913. doi: 10.2196/66913 (PMC12587018; doi:10.2196/66913)
Supplement: Multimedia Appendix 4 [file games_v13i1e66913_app4.docx]

|  | MSD approach | Design Methodology | Evaluated Measures |
| --- | --- | --- | --- |
| Jansen-Kosterin et al [38], 2013 | Rehabilitation | *NA* | User Experience: Usability, satisfaction, motivation, gaming experience |
|  |  |  | Training: Motor skills and clinical changes, physical condition |
| Idriss et al [39], 2017 | Rehabilitation | 3D Model Design, Physics Modeling, Scenario Design, Implementation | Usage Acceptability: Attractiveness of environments, clarity of instructions |
| France and Thomas [40], 2018 | Rehabilitation | *NA* | Effectiveness: evolution pain, disability, pain/injury anticipation, and number of lumbar flexions |
| Husna et al [41], 2025 | Rehabilitation | Technological feasibility | User Experience: Usability, gaming experience |
| Greuter and Tepe [42], 2013 | OHS awareness | *NA* | User Experience: Fun, engagement, success, control, motivation, feedback, usability, and difficulty |
|  |  |  | Learning: Academic test pre- and post-test, control group vs experimental group |
| Lanzotti et al [43], 2019 | OHS awareness | Participatory (design choice and evaluation) | Usability: Effectiveness, efficiency, satisfaction |
|  |  |  | Learning: Experimental group results in the game vs control group written multiple-choice questions |
| Pietrafesa et al [44], 2021 | OHS awareness | Co-design (needs analysis, ideation, evaluation) | *Not documented* |
| Rapp et al [45], 2019 | OHS awareness | Participatory | *NA* |
|  |  | Content Specification: Pedagogical content, game features, and usage context |  |
| Rebelo and Filgueiras [46], 2012 | OHS awareness | User Centered Design: Definition of user needs, objectives, and technology, prototype | *NA* |
| Kuipers et al [47], 2016 | MSD prevention | Iterative, collaborative | Engagement: Number and duration of sessions |
|  |  | User Centered Design: Definition of user needs, objectives, and technology, prototype evaluation, and field study | Training: Result tracking |
| Rodrigues et al [48], 2018 | MSD prevention | Game Concept Definition: Analysis, design, implementation, and testing | Usability/Satisfaction: Relevance of the game, ease of use, effectiveness of exercises, perceived well-being, overall satisfaction |
| Sisto et al [49], 2018 | MSD prevention | *Not documented* | *NA* |
| Intipanya et al [50], 2025 | MSD prevention | *NA* | Effectiveness: Adherence to procedure, perceived cervical discomfort, incidence of cervical pain, extent of active cervical movement, pain intensity, disability, quality of life and ability to work |
| Valdivia et al [51], 2017 | MSD prevention | Techno-centric approach | Engagement: Immersion, presence, flow, absorption |
| Stranick and Lopez [52], 2022 | MSD prevention | *NA* | Usability |
